# Speech intelligibility outcomes in maxillectomy patients rehabilitated with obturators prostheses: A prospective study

**DOI:** 10.6026/973206300221425

**Published:** 2026-03-31

**Authors:** Pritha Negi, Deeksha Kallala, Sunil Kumar Gulia, Raghavendra Sumanth Phani Challa, Rigzin Motup, Abhishek Gaur, Rahul Tiwari, Heena Dixit Tiwari

**Affiliations:** 1 Department of Prosthodontics and Crown & Bridge, K. M. Shah Dental College and Hospital, Sumandeep Vidyapeeth, Vadodara, Gujarat, India; 2 Department of quality management, Ryan White Quality Management Coordinator, Sixteenth Street Community Health Centers, Milwaukee, Wisconsin, USA; 3 Department of Oral and Maxillofacial Surgery, SGT University, Gurugram, Badli, Jhajjar, Haryana, India; 4 Department of Dentistry, AEGD Program, NYU Langone Health and Chief Dental Officer, Holyoke health center, 230 Maple Street, Holyoke, Massachusetts, USA; 5 Department of Prosthodontics and Crown & Bridge, Indira Gandhi Government Dental College, Jammu, Jammu & Kashmir, India; 6 Department of Periodontology, Seema Dental College & Hospital, Rishikesh, Uttarakhand, India; 7 Department of Dental Research Cell, Dr. D. Y. Patil Dental College & Hospital, Dr. D. Y. Patil Vidyapeeth (Deemed to be University), Pimpri, Pune, Maharashtra, India; 8 Department of blood cell, Programme Officer, Blood Cell, Commissionerate of Health and Family Welfare, Government of Telangana, Hyderabad, Telangana, India

**Keywords:** Speech quality, obturators, prosthetic therapy, maxillectomy, malignant tumor

## Abstract

Maxillectomy results in disruption of palatal continuity and oral-nasal separation, leading to significant impairment of speech intelligibility and communication that requires effective rehabilitative intervention. Hence, this prospective study assessed the speech quality of 30 patients recovering from maxillectomy who were fitted with prostheses for the obturators at baseline, 3 and six months post- insertion. The scores of intelligibility reported by patients and blinded evaluation ratings of evaluators were statistically significant improvements over time. Scores for the average patient increased by 2.03 + 0.45 at initial assessment to 4.23 + 0.57 after 6 months. Evaluation scores increased from 11.87 ± 0.40 → 3.32 ± 0.48 → 4.10 ± 0.46. Thus, we show that obturators prosthetic therapy leads to significant improvements in speech quality in the first few months of follow-up.

## Background:

Maxillectomy is the most common surgical procedure to treat malignant neoplasms of the maxilla and aggressive benign lesions, generating surgical defects that involveloss of palatal and oral-nasal continuity [1]. Palatal closure insufficiency leads to decreased nasal resonance and consonant articulation disorders resulting in hypernasal, culminating innasal air emission and poor speech intelligibility [2]. Obturator prosthesis has become commonly used for the re-establishment of palatal continuity to allow better generation of intraoral pressure and phonetic sound especially in caseswhere surgical reconstruction is impractical [3]. Advancements in the field of digital planning and design have significantly improved obturator fabrication accuracy and functionalfitting [4]. In addition, verified patient-reportinstruments have indicated the beneficial role of obturator prostheses for speech and quality-of-life outcomes following maxillectomy [5]. Therefore, it is of interest to report and evaluate longitudinal changes in speech intelligibility among maxillectomy patients rehabilitated with obturators prostheses using validated subjective and objective assessment tools.

## Materials and Methods:

This was a prospective observational study which was carried out betweenJune 2024 and January 2025 in a tertiary dental teaching hospital. In the present investigation adult patients ( ≥ 18 years) who had undergone unilateral partial or total maxillectomyand scheduled for obturators reconstruction were prospectively included. Excluded criteria wereas follows: disease recurrence, cognitive impairment for answering the questionnaires and loss of follow-up. Speech intelligibility was measured at baseline(pre-obturators), 3 and 6 months post- insertion with a structured speech intelligibility questionnaire rated on a 5 point extended Likert scale. Quality of speech perception testing was carried out by three trained assessorsin deterministic conditions by means of a 0-5 scale and standard text. Study Design Demographics, defect type and obturators (provisional versusdefinitive) were documented. Statistical analyses were conducted using SPSS for WINDOWS v26 and repeated measures ANOVA, with time as the within-subjects factor and P < 0.05 as the significance level.

## Results:

All patients were followed up for 6months. The average age was 52.4± 11.6 years and there was a male predominance (63.3%). Brown Class II was the most common (60%), followed by Brown Class I (26.7%) and Brown Class III (13.3%). Definitiveobturators were employed in 66.7% of the patients (Table 1 - see PDF). Patient-reported intelligibility scores demonstrated a significant improvement from baseline (2.03 ± 0.45) to 3 months (3.47 ±0.51) and 6 months (4.23 ± 0.57) post-implantation period (p <.001). Likewise, blinded evaluator classification increased from 1.87 ± 0.40 at baseline to 3.32 ± 0.48 at three months and to four.10 ± 0.46 at six months (p <.001) (Table 2 - see PDF). The change in median improvement between definitive obturators and interim prostheses wasslightly higher, but not significant (p = 0.071). In general, a gradual increase in speech intelligibility was notedwith time (Table 2 - see PDF).

## Discussion:

The results of this study demonstrate separately for the first time that obturator prosthesis rehabilitationwas associated with significant and sustained improvement in intelligibility up to 6 months following maxillectomy. Improvements were supported by estimation from both patient-reported outcomes and blinded evaluator ratings, consistent with the prior study that reported obturators prostheses improve resonance and consonant articulation by restoring palatalfunction [1, 2]. Obturator fit and functional results have been demonstrated to be increased by digital planning" / "CAD-CAM workflowplanning and digital design of the obturator, which indicates that complete digital workflows may increase patient adaptability and acceptance [4]. The increase in speech intelligibility at 3 months and the maintenance of a significant improvement at 6 monthsprobably captures learning and neuromuscular adaptation experienced with prosthesis use, published also observed in the literature of speech acoustics [4]. Hypernasality and nasal air emission continue to be the main factors of diminished intelligibility in maxillectomy patients; obturators help improve the pressure and airflow controlwithin the oral cavity, which in turn has shown to enhance phonemic discrimination [2, 5]. Subjective and objective speech assessment combined may enhance the evidencefor obturators effectiveness, as subjective patient-evaluation results and those of blinded evaluator ratings (speech-language experts) could reflect different features of functional rehabilitation [5]. Definitive obturator trended toward better improvement possibly because of improved retention andcontour, which was observed in comparative rehabilitation studies [6]. In contrast, the rehabilitation results ofmaxillectomy are a multifactorial outcome involving defect size, residual dentition and patient adjustment. Certain studies have highlighted the added value of speech therapy combined with prosthetic treatment, which wasn't standardized in this study but couldexplain a possible benefit of the changes observed [7]. In addition, implant- retained obturators have been demonstrated to be better retained and supported, despite that access toimplants is limited and not always universally possible [8, 9]. The established quality of life- for-the-obturators tool supports our finding that improvements in obturators functionhave a beneficial effect on social communication and functioning domains, with attainment of improved intelligibility [5]. Case reports have reported the use of precision attachments, as well as the development of a digitally manufactured obturatorstechnique-all indicative of new tendencies that might contribute to improve speech outcomes [7]. Such research could include long-termspeech function, objective oropharyngeal imaging and a comparison of patterns of therapy across different clinical areas [11 11-12]. Recent investigations have further demonstrated that structured prosthetic rehabilitation combined with speech assessment tools significantly improves articulation and resonance outcomes in maxillectomy patients undergoing obturator therapy. Additionally, contemporary prosthodontic and surgical literature highlights the role of advanced prosthetic design and multidisciplinary rehabilitation in optimizing functional speech recovery following maxillary defects [13, 14-15].

## Conclusion:

Maxillary obturator prostheses over a month period, significantly improve speech intelligibility between maxillectomy patients. Results both patient-reported andevaluator-rated scores improved progressively. These results reinforce obturators prosthetic rehabilitation as effective in improving speech functionfollowing maxillectomy.

## Advancement to knowledge:

This prospective study provides contemporary (2020-2026-aligned) longitudinal evidence demonstrating significant and progressive improvement in speech intelligibility following obturators prosthetic rehabilitation after maxillectomy, validated through both patient-reported and blinded evaluator-rated measures, thereby strengthening current evidence that functional prosthetic intervention contributes measurably to early speech recovery and quality-of-life restoration.
